# Effect of Occupational Exposure to Radar Radiation on Cancer Risk: A Systematic Review and Meta-Analysis

**DOI:** 10.31557/APJCP.2019.20.11.3211

**Published:** 2019

**Authors:** Ali Safari Variani, Somayeh Saboori, Saeed Shahsavari, Saeed Yari, Vida Zaroushani

**Affiliations:** 1 *Department of Occupational Health Engineering, Faculty of Health, *; 3 *Instructor of Biostatistics, Health Product Safety Research Center, *; 6 *Social Determinants of Health Research Center, Qazvin University of Medical Sciences, Qazvin, *; 2 *Department of nutrition, Lorestan University of Medical Sciences, Lorestan, *; 4 *Department of Epidemiology and Biostatistics, School of Public Health, Tehran University of Medical Sciences, *; 5 *Student Research Committee, Department of Faculty of Health, Shahid Beheshti University of Medical Sciences, Tehran, Iran. *

**Keywords:** Occupational cancer, occupational exposure, Radar, cancer, neoplasm, meta-analysis

## Abstract

**Objective:**

Microwave radiation is one of the most growing environmental workplace factors that exposes too many workers in the various workplaces. Regard to concerns about cancer incidence in these workers and lack of systematic or meta-analytic studies about this object, so, we conducted a meta-analysis to acquire an understanding of the association between cancer risk and occupational exposure to radar radiation.

**Methods:**

A systematic search was carried out on case-control, cohort and clinical control trial studies that published in the Cochrane Library, PubMed, ISI Web of Science, Scopus and Google scholar databases that accomplished from March 2017 to March 2018 and updated on 30 September, 2018 in English and Persian articles without time limit in publication date. Keywords were selected based on PICO principle and collected from MeSH database. After removal of duplicated studied, taking into inclusion and exclusion criteria, the process of screening was carried out and data were extracted after preparation of the full text of included articles. Article collection was completed by manually searching for a reference list of eligible studies. For quality assessment of included studies, Newcastle-Ottawa scale was used.

**Results:**

a total of 533 studies was found in the first step of literature search, only 6 were included with 53,008 sample size according to inclusion and exclusion criteria. Estimated pooled random effects size analysis showed no significant increasing effect of occupational exposure to radar radiation on mortality rate (MR=0.81, 95%CI: 0.78, 0.83) and relative risk (RR=0.87, 95%CI: 0.75, 0.99, P <0.0001) of cancer with a significant heterogeneity between the selected studies.

**Conclusions:**

In conclusion, the results of this meta-analysis study have shown no significant increase in overall mortality ratio and cancer risk ratio from occupational exposure to the radar frequency of workers. But, these results are not conclusive. As regards to some limitation such as fewer numbers of included studies, lack of data about exposure characterizations and demographic characterizations in this meta-analysis, this result is not certain and conclusive. It is recommended to conduct future studies.

## Introduction

Microwave (MW) radiation became one of the most significant and fastest growing environmental factors due to intensive development of communication technologies during the last decades. In microwave spectra, radar frequency with 1-300 GHz range has varied applications such as satellite, communications, military, Network, navigation, air-traffic Control, navigation, marine and weather. Therefore, many workers are exposed to these waves (Yakymenko et al., 2011; Zaroushani et al., 2014; Zaroushani et al., 2016). Pulsed MW, which generally affect only certain groups of military or service staff or population living nearby. Concerns have been raised about the safety of the microwave emissions of radars. Radars are detection systems which use MW to determine both moving and fixed objects like aircraft, ships, missiles, etc. Depending on the tasks they use different frequencies of MW (Yakymenko et al., 2011). Some common types of radars encountered with daily life include: Air traffic control radars, Weather radars, military, marine and Speed control radars. Therefore, too many workers are exposed to radar radiation (Zaroushani et al., 2014). There are many studies on exposure assessment and biological effects of microwave radiation (Khavanin et al., 2008; Zaroushani et al., 2014; Zaroushani et al., 2016; Zaroushani et al., 2016).

Previously, many case-controls, reviews and epidemiological studies were conducted on a wide range of electromagnetic radiation to investigate incidence cancers such as testicular, leukemia, brain tumor and other cancers (Davis and Mostofi,1993; Savitz, 1993; Grayson, 1996; Ofermerimsky et al., 1996; Goldsmith, 1997; Hardell et al., 1998; Richter et al., 2000; Baumgardt et al., 2002; Elwood, 2003; Clapp et al., 2005; Walschaerts et al., 2007; McGlynn and Trabert 2012). Also, some studies showed that radar frequency could have an adverse health effects that are divided into thermal and non-thermal effects (Zaroushani et al., 2014) cancer is one of the non-thermal adverse health effects due to occupational exposure to microwave and radar radiation. Brain cancer in the police officer (Gu et al., 2011) cancer stimulation in cancer cells (Yakymenko et al., 2011), significant elevation in non-lymphocytic leukemia and little effect on mortality in US Navy veterans (Groves et al., 2002) were some previous considered studies. Substantial military and occupational data indicate a significant effect of pulsed microwaves on cancer grows and other pathological conditions in workers (Yakymenko et al., 2011).

International Agency for Research on Cancer (IARC) has classified radio frequency electromagnetic fields as possible carcinogenic to humans (Group 2B), based on an increased brain cancer risk that associated to wireless phone use (Gaudin, 2011). However, because of differences in the design and extraction of these studies, their results are difficult to interpret. 

Regard to previous studies that showed there are controversial reports on cancer incidence of workers that occupationally exposed to radar radiation and lack of systematic or meta-analytic studies about this important object, so, we conducted a meta-analysis to acquire an understanding of the association with cancer risk and occupational exposure to radar radiation.

## Materials and Methods

*Search strategy and study selection*

The present study was carried out based on PRISMA guidelines. A comprehensive search strategy was focused by two independent researchers (VZ and ASV) on English articles without time limits in publication date, that collected from March 2017 to march 2018 and update on September 2018 in the Cochrane Library, PubMed, Scopus, Google Scholar, and ISI Web of Science Database with the use of following search terms (based on PICO principle): (worker OR technician OR occupation OR military OR airline OR navy OR police officer OR Weather) AND (occupational exposure OR workplace OR long-term exposure OR exposure OR radar OR microwave OR wireless OR high frequency range OR radio frequency OR radiation OR electromagnetic) AND (control group, cohort OR prospective OR retrospective OR follow-up OR randomized control trial OR case-control) AND (cancer OR malignant OR melanoma OR metastatic OR non-thermal effect OR biological effect OR health effect OR Adverse Effect OR risk factor OR Sarcoma OR tumor or leukemia OR neoplasm OR Carcinoma OR Hepatoma OR lymphoma OR mortality) as single or complex terms in titles, abstracts and keywords, that was reported the risk estimate indicators such as Standardized Incidence Ratio (SIR), Odds Ratios (OR), Relative risk (RR) and Mortality Ratio (MR) were collected as eligible studies. This systematic research was completed by a manual search for the reference list of eligible studies.

*Inclusion and exclusion criteria*

the inclusion criteria for selection studies were in design of case–control, cohort and randomized control trial studies, with a control group and referring to the association between occupational exposure to radar radiation and all types of cancer in workers.

The exclusion criteria were as follows: 1) studies without control group 2) reviews, case and field studies, 3) studies with inhumane population such as in vitro, in vivo and animal studies. 4) Studies that radar frequencies were out of considered ranges (1-300 GHz) .5) studies with other occupational or non-occupational carcinogenic risk factors (such as solvents, workplace air pollution, environmental air pollution, smoking and etc.).

*Data extraction and quality assessment*

After removal of duplicated studied, two reviewers independently according to the pre-specified selection criteria that describe in eligible study section screened the title and abstract of the literature for rebalance of the topic. Then, data extraction was done. Also, any disagreements were resolved by consensus among reviewers. For quality assessment of included studies, Newcastle-Ottawa Scale (NOS) was used (Wells et al., 2009; Li et al., 2008). This scale with two different tools for case-control and cohort studies scores, articles for selection, comparison and exposure/outcome assessments. The following information about selected articles were extracted: first author, origin of country, publishing year, gender, population aging, study design, sample size, follow-up period, workplace title, Relative risk (RR), odds ratio (OR), Mortality Ratio (MR).

*Data synthesis and Statistical analysis*

All statistical tests were conducted with STATA software package (version 12.0, College Station, TX) (Mantel and Haenszel, 1959; DerSimonian and Kacker, 2007). Odds Ratio, relative risk and Mortality Risk Ratio with their 95% confidence interval (CI) were used to assess the strength of association between occupational exposure to radar radiation and various cancer risks. Existence of heterogeneity was tested by Cochrane’s Q-test at P < 0.05 level of significance and the percentage of heterogeneity among studies was calculated by using I^2^ test. The random effect model analysis was used for estimating pooled effect size. Publication bias was evaluated by using funnel plot and beg test.

## Results

*Search result and study selection*

**Figure 1:**
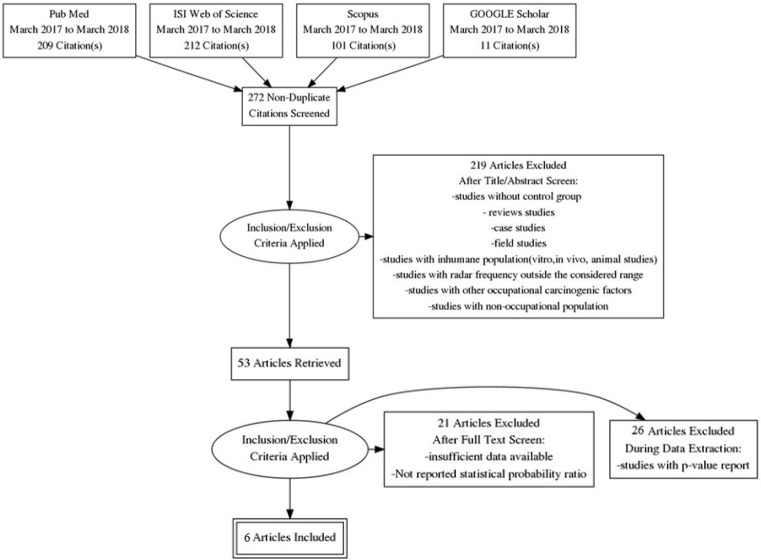
PRISMA Flow Diagram to Represent the Flow of Articles Reviewed in the Course of this Meta-Analysis

A total number of 533 studies were found in the first step of literature search in PubMed, Scopus, Google scholar and ISI Web of Science databases. After removal of duplicated references, 272 studies were included in the title, keywords and abstract screening. Then, 219 studies were excluded since they did not meet inclusion criteria. Hence, 54 studies were selected for the eligibility assessment. At the end of the selection process and after the quality assessment, 6 studies remained in the meta-analysis that including 3 case-control studies (Grayson 1996; Baumgardt et al., 2002; Walschaerts et al., 2007) and 3 cohort studies (Groves et al., 2002; Degrave et al., 2009; Dabouis et al., 2016) with no randomized control trial study. A manual search of the reference lists added no more articles in this meta-analysis. Flowchart of study selection for inclusion is shown in Figure 1.

The included studies in the current meta-analysis were carried out from 1993 to 2016 in various countries USA (Grayson, 1996; Groves et al., 2002), Germany (Baumgardt et al., 2002; Degrave et al., 2009), France (Walschaerts et al., 2007), Belgium/France (Dabouis et al., 2016) with 53,008 sample size and range of ages 15–69 years that examined the relationship between occupational exposure to radar radiation and cancer strength among workers. The Whole of the studies put the determination of cancers and risk estimates in military workers. All of the included studies were in the design of case-control (Grayson, 1996; Baumgardt et al., 2002; Walschaerts et al., 2007) and cohort studies (Groves et al., 2002; Degrave et al., 2009; Dabouis et al., 2016). Summarized characteristics of included studies in this meta-analyses base on mortality ratio, Odds Ratio and relative risk were presented in Tables 1 and 2. It is noticeable that the Groves et al reported both the relative risk and mortality ratio about cancer risk in workers who occupationally exposed to radar radiation (Groves et al., 2002). Tables 3 and 4 shows the quality of included studies assessed by Newcastle-Ottawa quality assessment scale was in the range of 7-9 stars.

*Meta-analysis*

**Figure 2.**
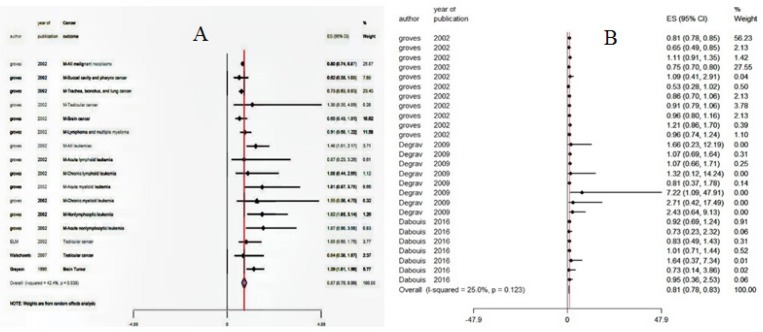
Forest Plot of Evaluation of Relative Risk (A) and Mortality Ratio (B) of Cancer in Workers who Occupationally Exposed to Radar Radiation

Results of meta-analysis of 4 studies with 16 arms (sharing data from RR and OR) showed no significant increase in cancer risk in workers who occupationally exposed to radar radiation (RR=0.87, 95%CI: 0.75, 0.99, P= <0.0001) with significant heterogeneity between studies (test for heterogeneity: P= 0.03 and I^2^=42.4%). A graphical display of estimated results, showed by the forest plot from individual included studies in this Meta-analysis (Figure 2 (A)). The left-hand column listed the characteristic of the studies, and the right-hand column is a plot of effect estimates with 95% confidence interval for each of the included studies.

Evaluation of the mortality risk was done and results of meta-analysis of 3 studies with 26 arms also, showed no significant increase in mortality rate (MR=0.81, 95%CI: 0.78, 0.83, test for heterogeneity: P= 0.12 and I^2^ = %25) in workers that exposed to radar radiation (Figure 2 (B)).

*Publication bias*

**Figure 3:**
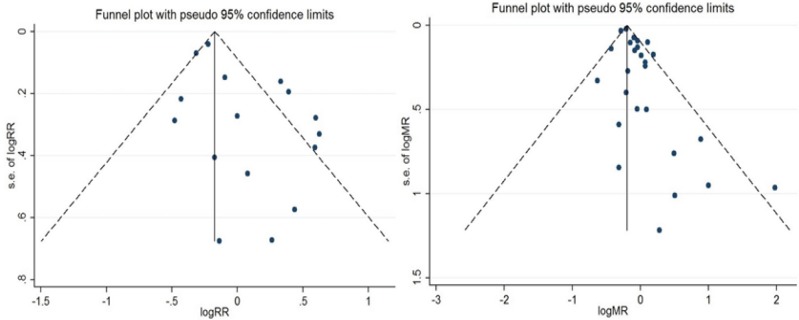
Funnel Plots for Relative Risk and Mortality Risk of Cancer in Workers who Occupationally Exposed to Radar Radiation

The Beg test showed no evidence for bias in the combined data from studies in the evaluation of the relative risk for cancer (P= 0.6) and mortality risk (P= 0.9) in workers who occupationally exposed to radar radiation. Funnel plots are shown in Figure 3.

**Table1:** Characteristic of Included Studies in Meta-analyses Base on Mortality Ratio (MR)

Study	Year	Origin Of Country	Study Design	Follow-Up Period	Sex	Sample Size	Workplace	Cancer Type	Mortality Rate	Lower-CI (95%)	Upper-95% CI
Groves (Groves, Page et al.2002)	2002	USA	cohort	1950-1997	Female-Male	40890	navy	All malignant neoplasms	0.81	0.78	0.85
								Buccal cavity and pharynx cancer	0.65	0.49	0.85
								Esophagus cancer	1.11	0.91	1.35
								Trachea, bronchus, and lung cancer	0.75	0.7	0.8
								Breast cancer	1.09	0.41	2.91
								Testicular cancer	0.53	0.28	1.02
								Brain cancer	0.86	0.7	1.06
								Lymphoma and multiple myeloma	0.91	0.79	1.06
								All leukemias	0.96	0.8	1.16
								Lymphocytic leukemia	1.21	0.86	1.7
								Nonlymphocytic leukemia	0.96	0.74	1.24
Degrav (Degrave, Meeusen et al. 2009)	2009	Germany	cohort	1963-1994	Male	7349	military	Lip, mouth and pharynx-	1.66	0.23	12.19
								Digestive organs and peritoneum-	1.07	0.69	1.64
								Respiratory and intrathoracic organs	1.07	0.66	1.71
								Bone, connective tissue, skin and breast	1.32	0.12	14.24
								Genitourinary organs -	0.81	0.37	1.78
								Lymphatic and hematopoietic tissue	7.22	1.09	47.91
								Eye, brain and nervous system -	2.71	0.42	17.49
								Other and unspecified sites-	2.43	0.64	9.13
Dabouis (Dabouis, Arvers et al. 2016)	2016	France	cohort	1975-2000	Male	1184	navy	mortality	0.92	0.69	1.24
								Lip, mouth and pharynx -	0.73	0.23	2.32
								Digestive organs and peritoneum-	0.83	0.49	1.43
								Respiratory and intrathoracic organsmortality	1.01	0.71	1.44
								Bone, connective tissue, skin and breast	NR*	NR	NR
								Genito-urinary organs -	NR	NR	NR
								Lymphatic and -	1.64	0.37	7.34
								Eye, brain and nervous system -	0.73	0.14	3.86
								Other and unspecified sites-	0.95	0.36	2.53

**Table 2:** Characteristic of Included Studies in Meta-analyses Base on Odds Ratio (OR) and Relative Risk (RR)

Study	Year	Origin Of Country	Study Design	Follow-Up Period	Gender	Sample Size	Workplace	Cancer Type	Out Come	Risk Ratio	Lower-CI (95%)	Upper- CI (95% )
Baumgardt-Elms C (Baumgardt-Elms, Ahrens et al. 2002)	2002	Germany	Case. Control	1995-1997	Male	1066	ships-airport-military- airplane	Testicular cancer	OR	1	0.6	1.75
Walschaerts (Walschaerts, Muller et al. 2007)	2007	FRANCE	Case. Control	2002-2005	Male	1029	Radar activity	Testicular cancer	OR	0.84	0.38	1.87
Grayson (Grayson 1996)	1996	USA	Case. Control	1970-1989	Male	1150	Us Air Force	Brain Tumor	OR	1.39	1.01	1.9
Groves (Groves, Page et al. 2002)	2002	USA	Cohort	1950-1997	Female-Male	40890	Navy	All malignant neoplasms	RR	0.8	0.74	0.87
								Buccal cavity and pharynx cancer	RR	0.62	0.35	1.08
								Trachea, bronchus, and lung cancer	RR	0.73	0.63	0.83
								Testicular cancer	RR	1.3	0.35	4.89
								Brain cancer	RR	0.65	0.43	1.01
								Lymphoma and multiple myeloma	RR	0.91	0.68	1.22
								All leukemias	RR	1.48	1.01	2.17
								Acute lymphoid leukemia	RR	0.87	0.23	3.26
								Chronic lymphoid leukemia	RR	1.08	0.44	2.66
								Acute myeloid leukemia	RR	1.81	0.87	3.78
								Chronic myeloid leukemia	RR	1.55	0.5	4.75
								Nonlymphocytic leukemia	RR	1.82	1.05	3.14
								Acute nonlymphocytic leukemia	RR	1.87	0.98	3.58

**Table 3:** Quality Assessment of Studies Using Newcastle-Ottawa Scale for Cohort Studies

Study, Year	Selection				Comparability of cohorts (matched for)	Outcome			Total score
	Representativeness of exposed cohort	Selection of Non-exposed cohort	Ascertainment of exposure	Outcome not present at baseline		Assessment of outcome	Sufficient follow-up duration	Adequate follow-up	
Groves FD,2002	*	*	*	*	** (age, Sex, Job title)	*	*	*	9
Degrave E,2009	*	*	*	*	** (age, sex, job title,)	*	*	*	9
Dabouis V, 2016	*	*	*	*	** (age, sex, job title,)	*	*	*	9

**Table 4:** Quality Assessment of Studies Using Newcastle-Ottawa Scale for Case-control Studies

Study, Year	Selection				Comparability of cases and controls (matched for)	Exposure			Total Score
	Adequate definition of cases	Representativeness of cases	Selection of controls	Definition of controls		Ascertainment of exposure	Same method of ascertainment for cases and controls	Non-response rate	
Elms CB,2002	*	*	*	*	** (age, sex, region, cancer type, job title)	*	*	-	8
Walschaerts M, 2007	*	*	*	*	** (age, sex, region, cancer type, job title)	-	*	*	8
Grayson J K, 1996	*	*	*	*	** (age, sex, cancer type, job title)	-	*	-	7

## Discussion

The current meta-analysis was carried out to assess the strength of cancer among workers with occupational exposure to radar radiation in different types of study design by included 6 studies (3 case–control studies, 3 cohort studies and no RCT studies) with publishing from 1993 to 2016 and 53,008 sample size. The Whole of the studies put the determination of cancers and risk estimates in military workers. The great majority of studies reported risk estimates with 95% confidence interval. Pooled results, found no significant increase in the overall relative risk and mortality ratio of cancer among workers with occupational exposure to radar radiation in comparison to control group.

This seems to be the opposite of the previous meta-analysis that assessed the association between the microwave and radio frequency exposure and cancer risks in both environmental and occupational settings found an increased risk of morbidity and/or mortality from lymphoma, leukemia, melanoma and brain/CNS cancers, following exposure to microwave and radiofrequency (Atzmon et al., 2016). It is noticeable that previous meta-analysis focused on both environmental and occupational exposure in the worker and public population and it clearly did not determine the relationship between occupational radar exposure and cancer risk in the workplace (Peleg et al., 2018). Whilst our meta-analysis just focused on occupational exposure to radar radiation that lead to a small number of included studies that contained inclusion criteria. In addition, due to few included studies, we could not separate cancer categories along with individual cancer categories and conduct subgroup analysis. So, we have to combine all the results from included studies to make this meta-analysis. However, based on these meta-analysis results, it seems that occupational exposure to radar radiation could not statistically increase cancer risk in related workers. Therefore, it needs to continue case–control, cohort and RCT studies.

Also, some previous systematic review without meta-analysis and statistically pooled results, reported some studies that showed occupational exposure to microwave and radar radiation can enhance the risk of cancer (Yousif et al., 2010; Yakymenko et al., 2011).

Also, there was a significant heterogeneity between the included studies in this meta-analysis that showed occupational exposure to radar frequency range could not significantly increase in overall relative risk of cancer that is may be arising from a small number of included studies.

The IARC Monograph Working Group discussed and evaluated the available literature on the occupational exposures to radar and microwaves (Gaudin, 2011). In addition, based on differences in the design and execution of these studies, World Health Organization (WHO) declare that their results are difficult to interpret, so it proposes to conduct further studies particularly case-control studies (Hardell, 2017). However, based on pooled results in current meta-analysis, it seems that the carcinogenicity of radar radiation with occupational exposure in workers is not statistically certain.

As we know, microwave energy is not sufficient to break the chemical bonds in DNA directly, but adverse effects may be mediated by indirect mechanisms, such as generation of oxygen free radicals (Vrhovac et al., 2011; Yakymenko et al., 2011) or a disturbance in DNA-repair processes (Levitt and Lai, 2010; Deshmukh et al., 2013; Zhi et al., 2017). Observations to date suggest that oxidative stress and cancer are closely linked. Oxidative stress can lead to chronic inflammation, which in turn could mediate most chronic diseases, including cancer, via transformation of a normal cell to tumor cells, tumor cell survival, proliferation, more resistance, radio resistance, invasion, angiogenesis and stem cell survival (Reuter, Gupta et al. 2010). Reactive oxygen species (ROS) are involved in a wide variety of different cancers such as Leukemia (Sumi et al., 2010), Lymphoma (van de Wetering et al., 2008), Prostate (Khandrika et al., 2009) and melanoma (Fruehauf and Trapp, 2008). These cancers were reported in some studies that included in this meta-analysis (Reuter et al., 2010). The results of the current systematic review and meta-analysis, showed that occupational exposure to the radar frequency range did not significantly increase the mortality and cancer risk in exposed workers. However, it should be mentioned that these results are yielded by a few numbers of available studies with no report in occupational dose and time exposure to the radar frequency range. Also considering to some limitation such as fewer numbers of included studies, lack of data about exposure characterizations (exposure time, dose-response, average of exposure level) and demographic characterizations (average of age, average of experience, radar frequency range) it is better to continue further studies about this topic and future review studies include the congress publications without limitation in language. The results of this meta-analysis could be useful in radiation health policy for organizations that active in the field of non-ionizing radiation, especially radar radiation. In conclusion, the results of this meta-analysis showed no significant increase in overall mortality and cancer risk from occupational exposure to radar frequency in workers. As regards to mentioned limitations and few included studies in this meta-analysis, this result is not certain and conclusive. So, it is recommended to conduct future studies. 

In conclusion, the current meta-analysis was carried out to estimate occupational cancer risk among workers who occupationally exposed to radar radiation. In conclusion, the results of this meta-analysis study have shown no significant increase in overall mortality ratio and cancer risk ratio from occupational exposure to the radar frequency in workers. But these results are not certain and conclusive due to some limitation such as fewer numbers of included studies. In addition, as regard to lack of data about exposure characterizations and demographic characterizations in included studied, so it is recommended to conduct a future case–control, cohort and RCT studies in this topic. Also, the results of this meta-analysis could be useful to provide a preventive radiation program or radiation health policy for which organizations that active in cancer prevention and non-ionizing radiation especially radar radiation.
